# Maternal Diet Associates with Offspring Bone Mineralization, Fracture Risk and Enamel Defects in Childhood and Influences the Prenatal Effect of High-Dose Vitamin D Supplementation

**DOI:** 10.3390/nu16030405

**Published:** 2024-01-30

**Authors:** Min Kim, Pia E. Nørrisgaard, Nilo Vahman, Olivier N. F. Cexus, Paul A. Townsend, Jakob Stokholm, Klaus Bønnelykke, Bo Chawes, Nicklas Brustad

**Affiliations:** 1 COPSAC, Copenhagen Prospective Studies on Asthma in Childhood, Herlev and Gentofte Hospital, University of Copenhagen, 2820 Copenhagen, Denmark; min.kim@dbac.dk (M.K.); pia.norrisgaard@3shape.com (P.E.N.); nilo.vahman@dbac.dk (N.V.); stokholm@copsac.com (J.S.); kb@copsac.com (K.B.); chawes@copsac.com (B.C.); 2 Faculty of Health and Medical Sciences, University of Surrey, Guildford GU2 7XH, UK; o.cexus@surrey.ac.uk (O.N.F.C.); p.townsend@surrey.ac.uk (P.A.T.); 3 Department of Food Science, University of Copenhagen, 1958 Copenhagen, Denmark

**Keywords:** vitamin D, pregnancy diet, bone health, dental health

## Abstract

We previously demonstrated a beneficial effect of high-dose vitamin D in pregnancy on offspring bone and dental health. Here, we investigated the effect of maternal dietary patterns during pregnancy on the risk of bone fractures, bone mineralization and enamel defects until age 6 years in the offspring. Further, the influence of diet on the effect of high-dose vitamin D was analyzed in the COPSAC_2010_ mother–child cohort including 623 mother–child pairs. A weighted network analysis on FFQs revealed three specific maternal dietary patterns that associated (Bonferroni *p* < 0.05) with both offspring bone and dental health. The effect of prenatal high-dose (2800 IU/day) vs. standard-dose (400 IU/day) vitamin D on offspring bone mineral content (adjusted mean difference (aMD): 33.29 g, 95% CI: 14.48–52.09, *p* < 0.001), bone mineral density (aMD: 0.02 g/cm^2^ (0.01–0.04), *p* < 0.001), fracture risk (adjusted incidence rate ratio: 0.36 (0.16–0.84), *p* = 0.02), and enamel defects in primary (adjusted odds ratio (aOR): 0.13 (0.03–0.58), *p* < 0.01) and permanent molars (aOR: 0.25; (0.10–0.63), *p* < 0.01) was most pronounced when mothers had lower intake of fruit, vegetables, meat, eggs, sweets, whole grain, offal and fish. This study suggests that prenatal dietary patterns influence offspring bone and dental development, and should be considered in order to obtain the full benefits of vitamin D to enhance personalized supplementation strategy.

## 1. Introduction

Vitamin D is a steroid hormone crucially involved in bone and dental mineralization which begins in utero and continues into adulthood [1,2]. Increasing evidence links vitamin D deficiency during pregnancy to a higher risk of dental defects [3,4,5,6] and inadequate bone development in offspring [7,8]. Therefore, maintaining sufficient vitamin D levels at the prenatal stage is vital to prevent children’s stunting and promote healthy growth.

Vitamin D can be acquired in a number of ways, primarily through sun exposure. However, dietary intake e.g., fatty fish intake and supplementation, also plays a crucial role [9]. A number of studies have shown the effect of a high intake of vitamin D during pregnancy on improved offspring bone [10,11,12] and dental health [13]. We have also observed the beneficial effects of high-dose vitamin D supplementation during the third trimester of pregnancy on offspring bone and dental health outcomes until age 6 years in the Copenhagen Prospective Studies on Asthma in Childhood 2010 (COPSAC2010) [14,15]. The supplementation effects were more pronounced among certain children for bone outcomes, suggesting the possibility of a targeted prevention approach. More specifically, we found an improved effect on bone outcomes among mothers with low pre-supplementation levels of 25-hydroxyvitamin D (25(OH)D) and among mothers giving birth during winter when sun exposure was low [15]. This trend was also observed in a more recent study [16]. However, as 25(OH)D measurements require blood sampling and can fluctuate over time and season, we evaluated a potential targeted non-invasive approach utilizing comprehensive food frequency questionnaires (FFQs) covering a month of dietary intake reflecting early pregnancy dietary patterns.

We first determined dietary patterns by performing a weighted gene co-expression network analysis (WGCNA) [17] on the FFQ data to determine dietary patterns of the mothers in the population-based cohort, the Copenhagen Prospective Studies on Asthma in Childhood 2010 (COPSAC2010). We then tested its association with maternal 25(OH)D levels and bone and dental outcomes to determine the influence of prenatal diet on offspring health outcomes. Offspring were previously found to benefit from high-dose vitamin D supplementation [14,15]. We then looked at a potential synergetic effect of dietary patterns and prenatal vitamin D supplementation according to these studies, to determine whether it may enhance the effectiveness of vitamin D supplementation. Finally, we aimed to identify metabolic pathways influenced by each dietary pattern by investigating the maternal blood metabolome at gestation week 24.

## 2. Methods

### 2.1. Study Population

The COPSAC_2010_ is a mother–child cohort with 736 participating families. A subset of pregnant women (*n* = 623) was enrolled in a double-blinded randomized controlled trial (RCT) at pregnancy week 24, where they were randomized 1:1 to a daily dose of 2400 IU per day of vitamin D_3_ supplementation or a matching placebo tablet (Camette A/S, Denmark) until one-week postpartum (ClinicalTrials.gov: NCT00856947; EudraCT: 2008-007871-26). A detailed description of the study can be found in previous reports, including inclusion and exclusion criteria of the participants [18]. Additionally, all women were given 400 IU/d of vitamin D_3_ supplementation during pregnancy as recommended by the Danish National board of Health. Hence, the study was a dose comparison of 2800 IU/day (high-dose group, *n =* 315) vs. 400 IU/day (standard-dose group, *n =* 308). COPSAC_2010_ was conducted in accordance with the guiding principles of the Declaration of Helsinki and was approved by the Local Ethics Committee (H-B-2009-014, approved 23 February 2009), the Danish Data Protection Agency (2015-41-3696). Written and oral informed consent were obtained at enrollment of participants.

### 2.2. Measurements

#### 2.2.1. FFQ

Maternal dietary intake during mid-pregnancy was obtained by a comprehensive FFQ in the 24th week of gestation. The FFQ was semiquantitative and consisted of 43 food and beverage items covering the period of four weeks prior to the FFQ [19].

#### 2.2.2. DXA Scans at Age 6 Years

Whole-body dual-energy radiography absorptiometry (DXA) scans were performed with Lunar iDXA densitometer (GE Healthcare, Chicago, IL, USA) with Encore analysis software with children lying on their backs and were performed from head to toe in one movement lasting approximately 3 min. The DXA scan assessed body composition in terms of fat, muscle mass, bone mineral density (BMD) and bone mineral concentration (BMC). For this specific study, we only used the whole-body bone outcomes i.e., total BMD and total BMC values. An experienced specialist examined all scan data and validated the quality of each image as previously detailed [15].

#### 2.2.3. Bone Fractures

History of bone fractures was obtained during childhood until 31 January 2019, through a combination of interviews with parents and medical record checks. Fractures were defined by radiologically verified fractures of the larger long bones (clavicle, radius, ulna, tibia, fibula, femur, and humerus), excluding fissures (i.e., minor cracks) [15,20].

#### 2.2.4. Dental Examination at Age 6 Years

At the six-year visit, a dental examination was carried out to collect detailed information regarding each child’s dental health status. Enamel defects (molar incisor hypomineralization, MIH) were defined as the presence of hypomineralized enamel of systemic origin with demarcated opacities, post-eruptive enamel breakdown, atypical restorations, and/or extractions of molars due to MIH according to the European Academy of Pediatric Dentistry criteria [21]. Demarcated opacities with a diameter of less than 2 mm were not scored. Likewise, other enamel disturbances, e.g., hypoplasia and dental fluorosis, were not scored. Children with at least one affected permanent molar were considered to have enamel defects. In addition, children with demarcated opacities in second molars in the deciduous dentition were identified [14,22].

### 2.3. Maternal Blood Metabolomic Profile

During enrollment of the study at gestation week 24, plasma samples were collected from the mothers. Metabolic profiling on these plasma samples was carried out by Metabolon, Inc. (Durham, NC, USA) using its HD4 platform. A detailed description of the metabolomics protocol is provided in the Supplementary Materials Section S1 and has also been described previously [23]. In brief, metabolites with missingness ≥ 30% as well as unannotated metabolites were excluded. This resulted in a metabolic profile consisting of levels of 753 metabolites used for analyses.

### 2.4. Statistical Analysis

#### 2.4.1. Dietary Patterns Based on WGCNA on FFQ

WGCNA [17] was used to identify dietary patterns, i.e., diet modules defined as groups of food items with similarities in the amount of food intake based on the maternal week 24 FFQ. The correlation between each food item quantifies their interconnectedness and assigns them to coexpression modules. First, *hclust* function was employed for sample hierarchical clustering to detect outliers where three samples were removed. Food items were then clustered and highly correlated modules were merged and summarized by an eigenvector (first principal component score) for each participant. 

#### 2.4.2. Food Modules and Bone and Dental Outcomes

Associations between the food modules and bone and dental outcomes were investigated using multivariate regression models including sex, pregnancy interventions (fish oil and vitamin D), gestational length, season of birth, exposure to tobacco smoke in pregnancy, length of exclusive breastfeeding in days, age at daycare start and age at the six-year visit. For total BMC and total BMD, we additionally adjusted for age of DXA scan, height and weight [15]. For dental outcomes a logistic regression model was used while quasi-Poisson regression was used for fracture frequency and linear regression for bone outcomes. Based on the food modules significantly associated with at least one bone or dental outcome (Bonferroni *p* < 0.05), participants were stratified into two groups by the food module median values (low vs. high), and the effect of the vitamin D intervention on the outcomes was thereafter investigated in each group. We also performed interaction analysis between food module scores (low vs. high) and pregnancy vitamin D intervention against the clinical outcomes. Lastly, we investigated the association between each food module score and 25(OH)D levels measured at week 24 in pregnancy, i.e., before the vitamin D supplementation, adjusted for sample season.

#### 2.4.3. Food Modules and Maternal Blood Metabolomic Profiles

To assess the component of the modules, we performed regression analyses between food modules and metabolites adjusted for maternal age at the time of blood collection and sex of the child. If we found any associations between food modules and metabolites at Bonferroni *p* < 0.05 we additionally performed pathway enrichment analysis.

All analyses were carried out by R Studio (version 2021.09.2) and associations with *p* < 0.05 are reported in this study.

## 3. Results

We had FFQ information and performed the WGCNA approach on 623 pregnant women at week 24. Of these, 490 were also enrolled into the vitamin D RCT (standard-dose *n =* 250, high-dose *n =* 240) [18]. Fracture outcomes were available for 492 children, DXA scans at age 6 years were available for 323 children [15], evaluation of enamel defects in permanent molars was available for 362 children while evaluation of enamel defects in primary molars was available for 509 children. Table 1 shows characteristics of mothers who had FFQ completed at gestation week 24 as well as characteristics of their offspring at the six-year visit.

### 3.1. Dietary Patterns during Pregnancy and Offspring Bone and Dental Outcomes

**Figure 1:**
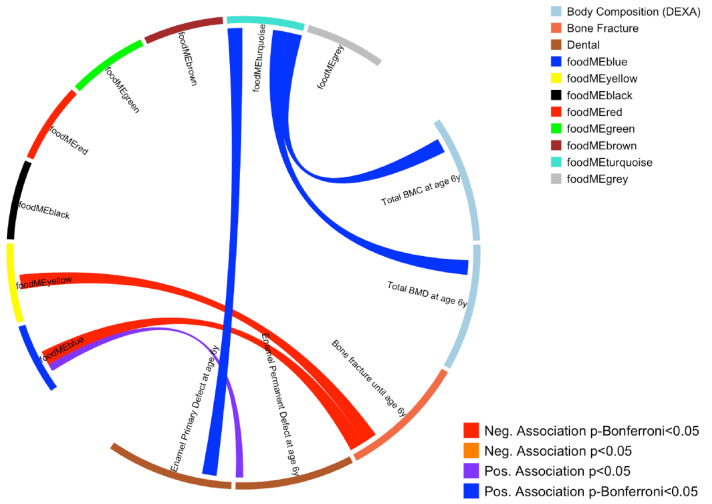
Circos plot showing association between maternal dietary patterns during pregnancy (food modules derived from FFQ WGCNA) and offspring bone and dental outcomes. Blue line represents a positive association at Bonferroni *p* < 0.05 level, purple line represents a positive association at nominal *p* < 0.05 level. Red line represents a negative association at Bonferroni *p* < 0.05 level.

**Figure 2:**
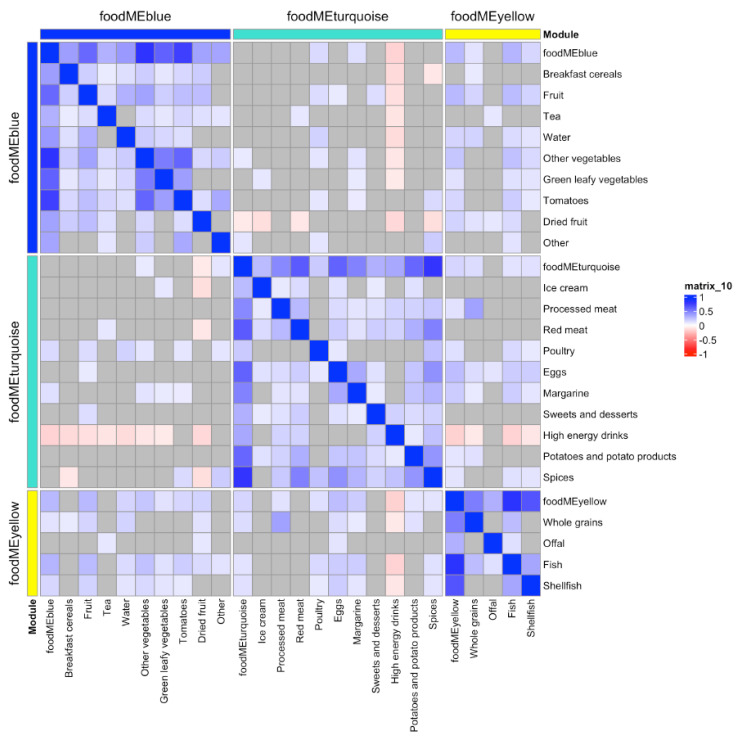
Heatmap of the three food modules which were found to associate with at least one bone or dental outcome and food items. Correlation key: blue represents positive Pearson’s correlations (*p* < 0.05), red represents negative Pearson’s correlations (*p* < 0.05) and grey presents non-significant correlations.

WGCNA on FFQ summarized 43 food items into eight modules, which also included a module containing uncorrelated food items (*grey* module, see Figure S1). An overview of these groups and a correlation heatmap showing the summary of the modules and food items is presented in Figure 1 and Table 1. Association between these eight modules and the bone and dental outcomes were then investigated, where three food module scores were found to associate with at least one outcome at Bonferroni *p* < 0.05 level (Figure 1). These three food module scores were *blue* (higher intake of fruits and vegetables), *turquoise* (higher intake of meat, eggs and sweets) and *yellow* (higher intake of whole grain, offal and fish). Food items included in these modules are illustrated in Figure 2 and Table S1.

After adjusting for potential confounding factors (sex, height, weight, pregnancy interventions (fish oil and vitamin D), gestational length, season of birth, exposure to tobacco smoke in pregnancy, length of exclusive breastfeeding in days, age at daycare start and age at the six-year visit), increased total BMC and BMD values at age 6 years were observed in children from mothers with higher *turquoise* module scores. A higher *turquoise* food module score was also associated with an increased risk of enamel defects in primary molars until age 6 years. Higher scores of *yellow* and *blue* modules were associated with reduced risk of bone fractures. All these associations were significant after multiple test corrections (Bonferroni *p* < 0.05 level) (see Figure 1 and Table S2).

Partial correlation analyses between 25(OH)D levels measured at week 24 in pregnancy at the same time-point as the FFQ was performed, and adjusted for sample season to account for the period of sunlight exposure, revealed positive correlations between 25(OH)D levels and the *blue* and *yellow* food module scores (r = 0.09, *p* = 0.03 and r = 0.12, *p* < 0.01, respectively). There was no significant correlation between 25(OH)D and the *turquoise* food module score (*p* > 0.05) (Figure S2).

### 3.2. Dietary Patterns and the Maternal Blood Metabolomic Profile

**Figure 3:**
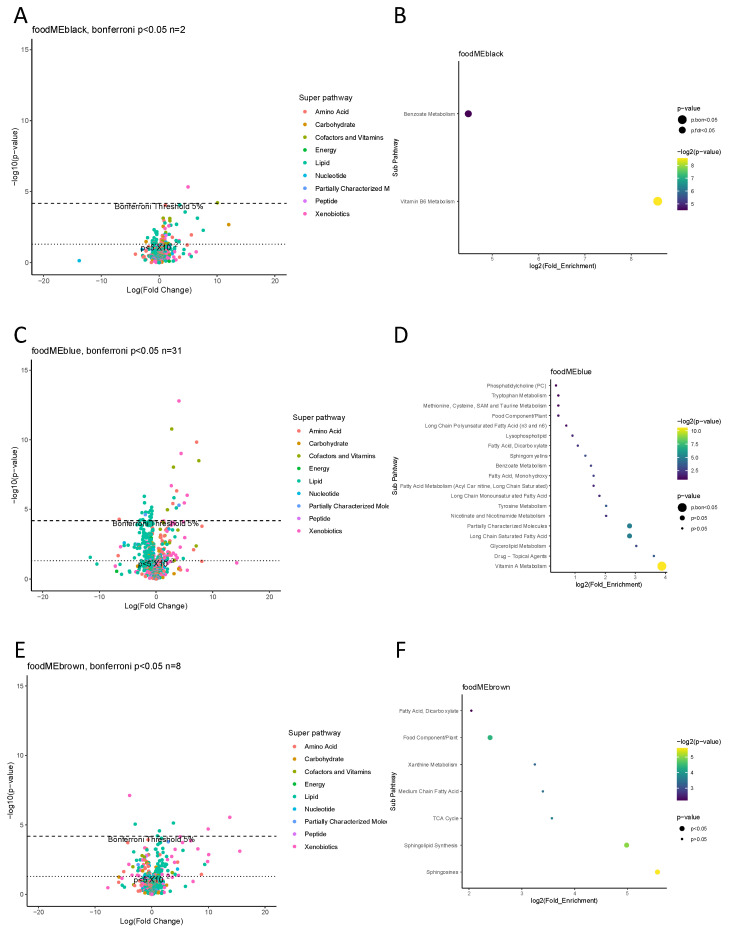

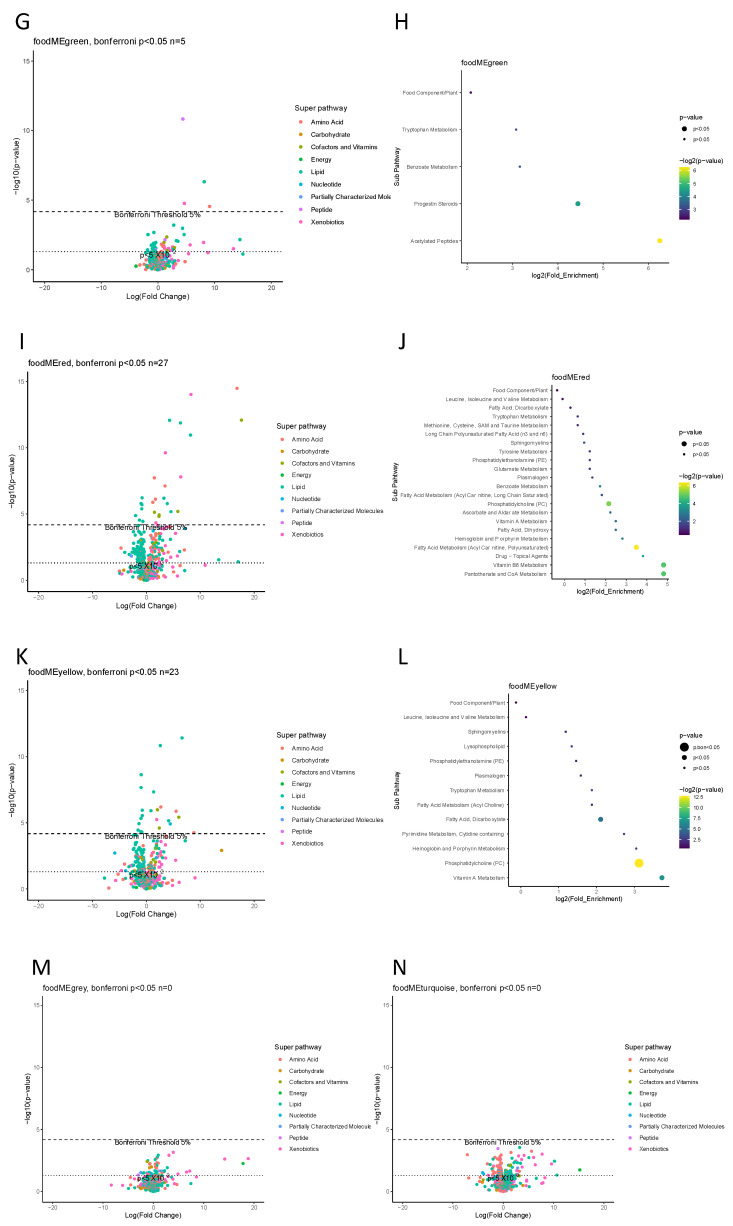
Maternal gestation week 24 metabolome vs. food modules. (**A**,**C**,**E**,**G**,**I**,**K**,**M**,**N**) Volcano plot showing a summary of linear regression between metabolite level and food module. Each dot represents a metabolite; different colors indicate metabolic super-pathways in which metabolites are involved. The x-axis indicates a change in metabolite level (per SD) with food module score, while the y-axis indicates association strength in terms of log10 of *p*-value. (**B**,**D**,**F**,**H**,**J**,**L**) Pathway enrichment analysis based on metabolites with Bonferroni *p* < 0.05. The y-axis indicates the metabolic sub-pathway name, while the x-axis indicates the logarithm of the enriched factor in each pathway. The bubble size and color indicate the *p* value. There was no enrichment analysis for two food modules, *grey* and *turquoise*, as no metabolite was found to associate at Bonferroni *p* < 0.05 level.

Six food modules were found to associate with at least one metabolite at Bonferroni *p* < 0.05 level, these food modules were *black*, *blue*, *brown*, *green*, *red* and *yellow* (Figure 3). Subsequent enrichment analysis showed *blue* food module affecting vitamin A metabolic pathway, *yellow* module affecting on phosphatidylcholine pathway and *black* module affecting vitamin B6 pathway at Bonferroni threshold *p* < 0.05. Other food modules showed no enriched metabolic pathways at the same threshold.

### 3.3. Effect of High-Dose Vitamin D Supplementation in Relation to Pregnancy Dietary Patterns on Offspring Bone and Dental Outcomes

We identified three food modules (*turquoise*, *yellow* and *blue)* associated with at least one bone or dental outcome at Bonferroni *p* < 0.05 level. These outcomes have previously been shown to benefit from vitamin D supplementation in pregnancy [14,15], hence, we stratified each of the maternal food modules into two groups using a median split (low vs. high) and investigated the effect of vitamin D supplementation according to these aiming to explore whether the intervention was more beneficial among certain groups of mothers dependent on their food intake in pregnancy.

Offspring from mothers who had low scores of the *turquoise* food module (i.e., lower intake of meat, eggs and sweets) benefited from high-dose vs. standard-dose vitamin D supplementation in both bone and dental outcomes. Total BMC and BMD values were higher (adjusted mean difference (aMD) = 33 g [95% confidence interval (CI): 14–52], aMD = 0.02 g/cm^2^ [0.01–0.04], *p*-values < 0.01), while the risk of enamel defects in permanent and primary molars were lower (adjusted odds ratio (aOR) = 0.25 [95% CI: 0.10–0.63], aOR = 0.13 [95% CI: 0.03–0.58], respectively, *p*-values < 0.01) when receiving high-dose vs. standard-dose vitamin D. Notably, high-dose vitamin D had no significant effect on offspring outcomes when mothers had high scores of this *turquoise* food module.

Offspring from mothers with low scores of the *yellow* food module (i.e., lower intake of whole grain, offal and fish) benefited from the high-dose vitamin D supplementation as their offspring had higher levels of BMC (aMD = 22 g [95% CI: 2–42], *p* = 0.04) and reduced risks of enamel defects in the primary molars (aOR = 0.29 [95% CI: 0.11–0.78], *p* = 0.01) compared with those receiving standard-dose vitamin D. Offspring from mothers with high food module scores did not benefit from the vitamin D supplementation on these two outcomes, or BMD, or frequency of bone fractures (*p* > 0.05). However, when analyzing the risk of enamel defects in permanent molars until age 6 years, high-dose vitamin D supplementation benefited children from mothers with both low and high *yellow* food module scores (aOR = 0.37 [95% CI: 0.16–0.87], *p* = 0.02; aOR = 0.35 [95% CI: 0.14–0.89], *p* = 0.03, respectively), suggesting an overall effect for this outcome independent of this specific dietary pattern in pregnancy. 

In offspring from mothers with low scores of the *blue* food module (i.e., lower intake of fruits and vegetables), high-dose vs. standard-dose vitamin D supplementation reduced the frequency of bone fractures in childhood (adjusted incidence risk ratio (aIRR) = 0.36 [95% CI: 0.16–0.84], *p* = 0.02) and reduced the risk of enamel defects in permanent molars (aOR = 0.27 [95% CI: 0.11–0.68], *p* < 0.01) at age 6 years. There were no specific effects on BMC, BMD and primary dentition enamel defects in these children. Offspring from mothers with high scores benefited from the intervention in terms of a reduced risk of enamel defects in primary molars (aOR = 0.30 [95% CI: 0.10–0.91], *p* = 0.03). Again, this suggests an overall effect for some clinical outcomes. 

**Figure 4:**
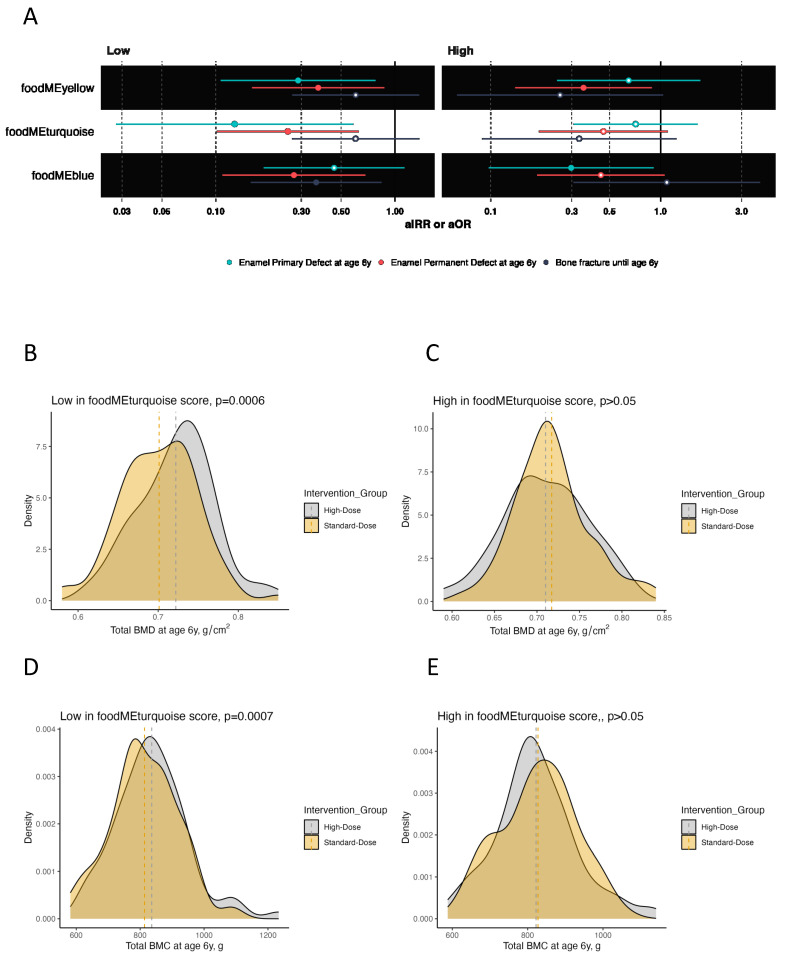

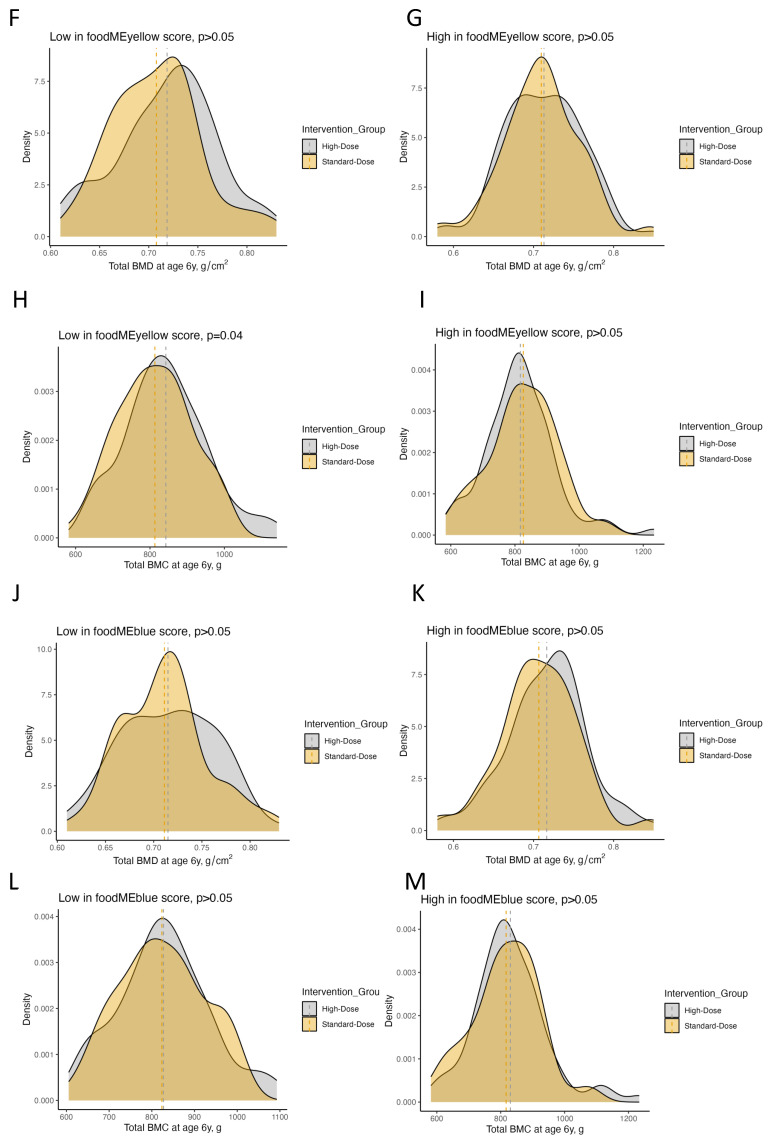
Plots showing the effect of high-dose vitamin D supplementation during pregnancy on offspring bone and dental outcomes at age 6 years stratified by low vs. high food module scores in for *turquoise*, *yellow* and *blue* food modules. (**A**) Forrest plot showing estimates for bone fracture frequency in terms of adjusted incidence rate risk (aIRR) and enamel defect status (no/yes) until age 6 years in terms of adjusted odds ratio (aOR). (**B**–**M**) Density plots showing the distribution of BMC and BMD in the high-dose and standard-dose vitamin D supplementation groups stratified by low vs. high food module scores. Abbreviations: BMC, bone mineral content; BMD, bone mineral density.

The results are illustrated as forest and density plots in Figure 4 and is outlined in Table 2.

We also tested for interactions and found significant interactions between *turquoise* food module score (low vs. high) and the vitamin D supplementation (high-dose vs. standard-dose) on total BMC and BMD (both *p* < 0.01) but not the other food modules and outcomes (Table S3).

## 4. Discussion

In this study, we investigated the relationship between mothers’ food intake patterns during early pregnancy by using a WGCNA approach and the risk of offspring bone and dental outcomes, showing that three of eight food patterns (modules) are associated with at least one of the outcomes. The most promising finding was the association between two maternal food patterns (*blue* and *yellow* modules) characterized by higher intake of fruits, vegetables, offal, fish and whole grain, and a reduced risk of offspring fractures. Further, we found that the effect of high-dose vs. standard-dose vitamin D supplementation in pregnancy on offspring bone and dental outcomes was more pronounced when the mothers had a low intake of these two food patterns (*blue* and *yellow* modules) as well as meat, eggs and sweets belonging to another food pattern (*turquoise* module). Lastly, maternal 25(OH)D level in pregnancy was positively associated with increased intake of foods from the *blue* and *yellow* food patterns, but not intake of foods from the *turquoise* food pattern, implying that dietary patterns provide additional information other than measuring 25(OH)D for determining the response to high-dose vitamin D intervention in pregnancy. These findings suggest that a targeted approach based on diet habits can maximize the beneficial effect of high-dose vitamin D supplementation in pregnancy on offspring bone and dental health, and ultimately reduce children’s stunting.

### 4.1. Interpretation

Previously, we found that the effect of high-dose vitamin D supplementation compared with a standard dose during the third trimester of pregnancy on offspring bone outcomes was more pronounced when giving birth during the dark months with low exposure to sunlight and when the mothers had initially low 25(OH)D levels. Our current study suggests that assessing dietary patterns could provide additional information compared to measuring 25(OH)D as a useful tool when searching for mothers whose children would have a more pronounced response to vitamin D supplementation. The dietary patterns to consider for a targeted approach were generally characterized by lower intake of vitamin D-rich food such as seafood (*yellow* module), fruits and vegetables (*blue* module) and eggs (*turquoise* module). We confirmed correlations between the food patterns and 25(OH)D levels in our data, except for the egg-containing food module (*turquoise*). The latter finding could be due to the inclusion of many other food items within the food module not correlating with 25(OH)D levels and suggests that assessing food intake provides additional information compared to measuring 25(OH)D. The correlations were adjusted for the season of blood sampling at week 24 in pregnancy to account for sunlight exposure. The FFQ reflected food intake 4 weeks prior, i.e., during early pregnancy, and might be considered as a tool in the development of a targeted precision prevention approach due to the associations with bone and dental outcomes in the offspring that also track into adulthood [24]. In such cases, a targeted vitamin D supplementation in pregnancy seems to hold beneficial effects on important disease outcomes such as fractures and dental diseases.

Higher intake of food items in the *turquoise* module during pregnancy was both associated with improved bone health outcomes in offspring and an increased risk of enamel defects in the primary molars. Where the latter is likely to be explained by a higher intake of sugar-rich substances such as ice cream, sweets, desserts and high-energy drinks, the improved bone outcomes could be explained by the high intake of e.g., eggs, which is a source of vitamin D. However, this food module did not associate with 25(OH)D levels, suggesting other important food sources. The dietary behavior of the mothers is likely to continue after birth, becoming a strong determinant for an offspring’s dietary habits also leading to a higher risk of diseases [25,26].

Here, we found that offspring from pregnant mothers with lower vitamin D-containing dietary patterns were more likely to benefit from the effect from high-dose vitamin D supplementation, which supports our previous findings of an effect on offspring bone outcomes among mothers giving birth during the dark months and with low 25(OH)D levels [15]. Many studies have investigated the effect of vitamin D supplementation, but the focus has been on adults [27,28], and in some cases infants [29], while vitamin D insufficiency/deficiency being defined by circulating 25(OH)D levels. While measurement of 25(OH)D involves an invasive blood sample test, FFQ is reasonably simple, inexpensive, time-efficient [30] and most importantly, it can reflect long-term dietary intakes [31,32]. This has led to a usefulness of FFQ for vitamin D assessment [31,33,34]. There have been studies looking at diets and bone health based on adult participants [35,36] but, to our knowledge, only one study has looked at dietary patterns during pregnancy on children’s bone health (offspring *n =* 216) [37]. This showed milk, fat and magnesium being predictive of spine BMD at age 16, but not total BMD. Here, spine BMD was not measured separately, hampering a direct comparison, but we found a similar food pattern (*Brown* module) containing milk and fat showing a trend towards a higher total BMC at age 6 years. This is possibly explained by the high intake of calcium that is the main part of hydroxyapatite (bone mineral).

To our knowledge, no study has examined the relationship between dietary patterns during pregnancy and offspring dental health, although there have been a few on children [38,39] and adult populations [40,41].

### 4.2. Strengths and Limitations

The strength of our study is the population-based prospective mother–child cohort with detailed information on maternal food intake comprising 4 weeks in early pregnancy in combination with 25(OH)D measurements and a double-blinded RCT. Further, we have detailed data on bone mineralization from whole-body DXA scans validated by an experienced specialist, longitudinally registered radiologically verified fractures and a thorough dental examination performed by a dental professional. The relatively large cohort is population-based, representing the general Danish population, which allows us to generalize our findings. Finally, to our knowledge, this is the first study to investigate beneficial effects of high-dose vitamin D supplementation in pregnant mothers with certain dietary patterns on children’s bone and dental outcomes and the first to associate these specific detailed food modules to an offspring’s risk of dental and bone outcomes. The study findings were limited by the lack of significant interaction for two of the three food modules with vitamin D supplementation, but this could be due to reduced statistical power. Previously, we also demonstrated the effects of fish oil intervention on bone mineralization. However, we adjusted all our analyses for this intervention.

## 5. Conclusions

This study suggests maternal food intake during early pregnancy to be important for offspring bone and dental development. Further, the assessment of dietary patterns in pregnancy might be a useful targeted non-invasive tool to personalize and optimize the effect of vitamin D supplementation in pregnancy for prevention of offspring bone and dental diseases.

## Figures and Tables

**Table 1:** Baseline characteristics stratified by the pregnancy vitamin D intervention. Results are mean (S.D.) for continuous variables. § categorical variable, *p* value derived from chi-square test. # Continuous variable, *p* value derived from student *t*-test, * *p* value < 0.05.

		All (*n =* 623)	Standard-Dose (*n =* 250)	High-Dose (*n =* 240)	*p* Value
#	25(OH)D level at gestation week 24 (nmol/l)	75.35 (24.78), *n =* 621	75.36 (25.61), *n =* 250	74.81 (23.76), *n =* 238	0.80
§	Fish oil intervention, No/Yes	307/295, *n =* 602	133/117, *n =* 250	127/113, *n =* 240	0.95
§	Offspring Sex, male/female	291/313, *n =* 602	123/127, *n =* 250	113/127, *n =* 240	0.64
§	Birth season, Spring/Summer/Fall/Winter	145/181/141/135/21, *n =* 602	73/51/57/69, *n =* 250	69/52/55/64, *n =* 240	0.99
#	Daycare start age, years	0.89 (0.23), *n =* 595	0.89 (0.27), *n =* 245	0.88 (0.20), *n =* 238	0.65
#	Gestational age at birth, weeks	278.93 (12.00), *n =* 621	278.91 (10.67), *n =* 250	280.17 (9.69), *n =* 239	0.17
#	Number of cigarettes mother smoked during 3rd trimester per day	2.14 (13.85), *n =* 606	3.25 (16.02), *n =* 250	1.80 (14.46), *n =* 240	0.30
#	Length of exclusive breastfeeding, days	104.06 (59.46), *n =* 599	108.57 (60.03), *n =* 247	102.27 (57.09), *n =* 239	0.0001 *
#	Offspring weight at age 6 y, kg	21.75 (2.96), *n =* 549	21.76 (2.87), *n =* 228	21.49 (3.01), *n =* 218	0.33
#	Offspring height at age 6 y, cm	118.44 (4.99), *n =* 549	118.32 (5.11), *n =* 228	118.03 (4.64), *n =* 218	0.53

**Table 2:** Pregnancy vitamin D intervention vs. bone and dental outcomes stratified by food module scores. Effect measures are adjusted mean differences for BMC and BMD outcomes, adjusted incidence risk ratio for the bone fracture outcome, and adjusted odds ratios for dental outcomes. Abbreviations: BMC, bone mineral content; BMD, bone mineral density.

	Clinical Outcomes	Food Module Low		Food Module High	
		Effect Measure [95 CI%]	*p* Value	Effect Measure [95 CI%]	*p* Value
foodMEyellow	Total BMC at age 6 y, g	22 [2–42]	0.04 *	10 [−7–27]	0.26
	Total BMD at age 6 y, g/cm^2^	0.01 [−0.01–0.02]	0.31	0.01 [−0.00–0.02]	0.08
	Bone fractures	0.60 [0.27–1.37]	0.23	0.26 [0.06–1.04]	0.06
	Enamel Permanent Defect at age 6 y	0.37 [0.16–0.87]	0.02 *	0.35 [0.14–0.89]	0.03 *
	Enamel Primary Defect at age 6 y	0.29 [0.11–0.78]	0.01 *	0.65 [0.25–1.72]	0.38
foodMEturquoise	Total BMC at age 6 y, g	33 [14–52]	0.0007 #	−6 [−23–12]	0.53
	Total BMD at age 6 y, g/cm^2^	0.02 [0.01–0.04]	0.0006 #	−0.01 [−0.02–0.01]	0.3
	Bone fractures	0.60 [0.27–1.36]	0.28	0.33 [0.09–1.24]	0.1
	Enamel Permanent Defect at age 6 y	0.25 [0.10–0.63]	0.003 #	0.46 [0.19–1.10]	0.08
	Enamel Primary Defect at age 6 y	0.13 [0.03–0.58]	0.008 #	0.71 [0.31–1.65]	0.43
foodMEblue	Total BMC at age 6 y, g	10 [−8–28]	0.28	16 [−3–35]	0.10
	Total BMD at age 6 y, g/cm^2^	0.01 [−0.01–0.02]	0.43	0.01 [−0.00–0.03]	0.06
	Bone fractures	0.36 [0.16–0.84]	0.02 *	1.09 [0.31–3.86]	0.90
	Enamel Permanent Defect at age 6 y	0.27 [0.11–0.68]	0.006 #	0.45 [0.19–1.06]	0.07
	Enamel Primary Defect at age 6 y	0.46 [0.18–1.13]	0.09	0.30 [0.10–0.91]	0.03 *

# passed Bonferroni, * nominal.

## Data Availability

The data presented in this study are available on request from the corresponding author.

## Supplementary Materials

The following supporting information can be downloaded at: https://www.mdpi.com/article/10.3390/nu16030405/s1, Section S1: Methods; Table S1: Food items in each food module derived from WGCNA; Table S2: Regression effect measures (after adjusting for covariates) and 95% confidence intervals (CI) describing the relationship between food module scores and clinical outcomes; Table S3: Summary of regression models on interaction term of food score (low vs. high) and pregnancy vitamin D intervention against the clinical outcomes; Figure S1: Heatmap of the 43 food items and 8 food modules derived from WGCNA; Figure S2: Figure showing correlation between 25(OH)D level at gestation week 24 and pregnancy food module scores.
