# Supplementary material for: Maternal Diet Associates with Offspring Bone Mineralization, Fracture Risk and Enamel Defects in Childhood and Influences the Prenatal Effect of High-Dose Vitamin D Supplementation
Source: Nutrients. 2024 Jan 30;16(3):405. doi: 10.3390/nu16030405 (PMC10856831; doi:10.3390/nu16030405)
Supplement: Supplementary file 1 [file nutrients-16-00405-s001.zip › nutrients-2821936-supplementary.pdf]

S1. Methods

Sample Preparation: Metabolomic profiling on EDTA plasma samples were prepared using the automated MicroLab STAR® system from Hamilton Company. Recovery standards in methanol were added followed by vigorous shaking for 2 min (Glen Mills GenoGrinder 2000) followed by centrifugation. The resulting supernatant extract was divided into five fractions: two for analysis by two separate reverse phase (RP) tandem mass-spectrometry (MS/MS) methods with positive ion mode electrospray ionization (ESI), one for analysis by RP MS/MS with negative ion mode ESI, one for analysis by hydrophilic interaction chromatography (HILIC) MS/MS with negative ion mode ESI, and one sample was reserved for backup. Samples were dried under nitrogen gas TurboVap® (Zymark) overnight prior to analysis.

Metabolic profiling: Dried samples were reconstituted in starting mobile phase solvents for each method. Samples were analyzed using ACQUITY Ultra-Performance Liquid Chromatography (UPLC) (Waters, Milford, USA) with Q Exactive™ Hybrid QuadrupoleOrbitrap™ mass spectrometer interfaced with heated electrospray ionization (HESI-II) source (ThermoFisher Scientific, Waltham, Massachusetts, USA). The sample extracts were reconstituted in solvents compatible to each of the four LC-MS methods utilized.

For RP in positive ESI mode optimized for more hydrophilic compounds, the extract was gradient eluted from a C18 column (Waters UPLC BEH C18-2.1x100 mm, 1.7 μm) using water and methanol, containing 0.05% perfluoropentanoic acid (PFPA) and 0.1% formic acid (FA). For RP in positive ESI mode optimized for more hydrophobic compounds, the extract was gradient eluted from the same C18 column using methanol, acetonitrile, water, 0.05% PFPA and 0.01% FA and was operated at an overall higher organic content. For RP in negative ESI mode, a separate dedicated C18 column was utilized where the basic extracts were gradient eluted from the column using methanol and water, both with 6.5 mM Ammonium Bicarbonate at pH 8. For HILIC in negative mode, HILIC column (Waters UPLC BEH Amide 2.1x150 mm, 1.7 μm) using a gradient consisting of water and acetonitrile with 10mM Ammonium Formate, pH 10.8, was utilized. The MS/MS setting involved MS and data-dependent MSn scans using dynamic exclusion, scan range between 70-1000 m/z, at mass resolution 35,000.

Data Extraction and Compound Identification: Raw data was extracted, peak-identified and QC processed using Metabolon's hardware and software. These systems are built on a web-service platform utilizing Microsoft's .NET technologies, which run on high-performance application servers and fiber-channel storage arrays in clusters to provide active failover and load-balancing. Compounds were identified by comparison to library entries of purified standards or recurrent unknown entities. Metabolon maintains a library based on authenticated standards that contains the retention time/index (RI), mass to charge ratio (m/z), and chromatographic data (including MS/MS spectral data) on all molecules present in the library. Furthermore, biochemical identifications are based on three criteria: retention index within a narrow RI window of the proposed identification, accurate mass match to the library +/- 10 ppm, and the MS/MS forward and reverse scores between the experimental data and authentic standards. The MS/MS scores are based on a comparison of the ions present in the experimental spectrum to the ions present in the library spectrum. While there may be similarities between these molecules based on one of these factors, the use of all three data points can be utilized to distinguish and differentiate biochemicals. More than 4,500 commercially available purified standard compounds have been acquired and registered into LIMS for analysis on all platforms for determination of their analytical characteristics.

Data processing pipeline: A data normalization step was performed to correct variation resulting from instrument inter-day tuning differences by registering the medians to equal one and normalizing each data point proportionately. Further normalization was used to correct for analyzed sample volume variation. Median relative standard deviation (RSD) from quality control samples (pooled matrix samples) were determined to evaluate instrument variability (median RSD=7%) and overall process variability (median RSD=10%). We calculated missingness across all samples for each metabolite, metabolites with missing ≥ 30% as well as unannotated metabolites were excluded. Missing values were imputed as half the minimum value across all samples for each metabolite. This resulted in a metabolic profile consisting of levels of 753 metabolites and subsequently used for further analyses.

Table S1. Food items in each food module derived from WGCNA. Arrows represent positive or negative correlation between food item and its food module. Presence of no arrow represents correlation at p>0.05. Values in brackets indicate median value (g/day) for each food item when the food module the food item belongs to is split by food module median value (low *vs.* high).

MEblack	MEblue	MEbrown	MEgreen	MEgrey	MEred	MEturquoise	MEyellow
↑Vegetable fats (3.46)	↑Breakfast cereals (10.71)	↑Low fat dairy (615.71)	↑Vegetable juice (0.00)	↑Wine (0.00)	↑Nuts (4.29)	↑Ice cream (7.14)	↑Whole grains (236.64)
↑Dressings (0.00)	↑Fruit (405.09)	↑High fat dairy (32.72)	↑Soy products (0.07)	↑Beer (0.00)	↑Beans, lentils (0.00)	↑Processed meat (7.26)	↑Offal (0.00)
	↑Tea (150.00)	↑Refined grains	↑Canned fruit (0.00)	Low energy drinks		↑Red meat (33.37)	↑Fish (23.73)

MEblack	MEblue	MEbrown	MEgreen	MEgrey	MEred	MEturquoise	MEyellow
		(43.57)		(53.57)			
	↑Water(1,488.60)	↑Animal fats (7.50)		↑Alcoholic beverages (0.00)		↑Poultry (28.20)	↑Shellfish (0.00)
	↑Other vegetables (72.13)	↑Coffee (13.39)				↑Eggs (21.36)	
	↑Green leafy vegetables (0.85)	↑Snacks (7.02)				↑Margarine (9.78)	
	↑Tomatoes (22.28)	↑Fruit juice (28.73)				↑Sweets and desserts (68.17)	
	↑Dried fruit (11.61)	↑Fruit syrup and marmalade (36.23)				↑High energy drinks (8.93)	
	↑Other (15.94)	↑Cheese (10.11)				↑Potatoes and potato products (107.77)	
						↑Spices (2.49)	

Table S2. Regression effect measures (after adjusting for covariates) and 95% confidence intervals (CI) describing the relationship between food module scores and clinical outcomes. Effect measures are adjusted mean differences for BMC and BMD outcomes, adjusted incidence risk ratio for the bone fracture outcome, and adjusted odd ratios for dental outcomes.

Food Module	Clinical Outcomes	Adjusted effect measure [95% CI]	p-value
MEblack	Enamel Permanent Defect at age 6y	0.91 [0.67–1.24]	0.56
MEblack	Enamel Primer Defect at age 6y	0.96 [0.70–1.30]	0.78
MEblack	Bone fractures	0.96 [0.69–1.33]	0.82
MEblack	Total BMC at age 6y	6.20 [−0.19–12.60]	0.06
MEblack	Total BMD at age 6y	0.00 [−0.00–0.01]	0.07
MEblue	Enamel Permanent Defect at age 6y	1.32 [1.01–1.74]	0.04
MEblue	Enamel Primer Defect at age 6y	1.18 [0.88–1.57]	0.27
MEblue	Bone fractures	0.57 [0.39–0.85]	0.006
MEblue	Total BMC at age 6y	−1.67 [−8.19–4.85]	0.62
MEblue	Total BMD at age 6y	0.00 [−0.00–0.01]	0.52
MEbrown	Enamel Permanent Defect at age 6y	0.93 [0.69–1.25]	0.63

MEbrown	Enamel Primer Defect at age 6y	1.18 [0.87–1.61]	0.28
MEbrown	Bone fractures	1.01 [0.76–1.34]	0.96
MEbrown	Total BMC at age 6y	7.24 [0.00–14.48]	0.051
MEbrown	Total BMD at age 6y	0.00 [−0.00–0.01]	0.06
MEgreen	Enamel Permanent Defect at age 6y	1.04 [0.68–1.60]	0.85
MEgreen	Enamel Primer Defect at age 6y	1.12 [0.68–1.85]	0.66
MEgreen	Bone fractures	0.64 [0.30–1.35]	0.24
MEgreen	Total BMC at age 6y	−2.95 [−12.66–6.76]	0.55
MEgreen	Total BMD at age 6y	−0.00 [−0.01–0.00]	0.55
MEgrey	Enamel Permanent Defect at age 6y	0.89 [0.67–1.20]	0.45
MEgrey	Enamel Primer Defect at age 6y	0.94 [0.67–1.32]	0.72
MEgrey	Bone fractures	1.16 [0.89–1.50]	0.28
MEgrey	Total BMC at age 6y	1.91 [−4.33–8.14]	0.55
MEgrey	Total BMD at age 6y	0.00 [−0.00–0.01]	0.24
MEred	Enamel Permanent Defect at age 6y	0.76 [0.51–1.14]	0.18
MEred	Enamel Primer Defect at age 6y	1.03 [0.81–1.30]	0.82
MEred	Bone fractures	0.52 [0.25–1.10]	0.09
MEred	Total BMC at age 6y	0.09 [−5.55–5.74]	0.97
MEred	Total BMD at age 6y	−0.00 [−0.01–0.00]	0.53
MEturquoise	Enamel Permanent Defect at age 6y	1.11 [0.80–1.53]	0.53
MEturquoise	Enamel Primer Defect at age 6y	1.56 [1.13–2.14]	0.006
MEturquoise	Bone fractures	0.74 [0.51–1.07]	0.11
MEturquoise	Total BMC at age 6y	10.89 [3.70–18.09]	0.003
MEturquoise	Total BMD at age 6y	0.01 [0.00–0.01]	0.009
MEyellow	Enamel Permanent Defect at age 6y	1.01 [0.78–1.30]	0.96
MEyellow	Enamel Primer Defect at age 6y	1.00 [0.73–1.36]	1.00
MEyellow	Bone fractures	0.48 [0.32–0.73]	0.001
MEyellow	Total BMC at age 6y	−2.47 [−8.89–3.95]	0.45
MEyellow	Total BMD at age 6y	−0.00 [−0.01–0.00]	0.76

Table S3. Summary of regression models on interaction term of food score (low *vs.* high) and pregnancy vitamin D intervention against the clinical outcomes. Effect measures are adjusted mean differences for BMC and BMD outcomes, adjusted incidence risk ratio for the bone fracture outcome, and adjusted odd ratios for dental outcomes. Abbreviations: BMC, bone mineral content; BMD, bone mineral density

	Clinical Outcomes	Food Module Low	
		Effect Measure [95CI%]	p-value
foodMEyellow	Total BMC at age 6y, g	−14.42 [−40.23–11.40]	0.27
	Total BMD at age 6y, g/cm^2	0.00 [−0.02–0.02]	0.88
	Bone fractures	0.48 [0.10–2.29]	0.36
	Enamel Permanent Defect at age 6y	1.01 [0.30–3.38]	0.99
	Enamel Primary Defect at age 6y	2.20 [0.56–8.63]	0.26
foodMEturquoise	Total BMC at age 6y, g	−34.31 [−59.69–8.93]	<0.01
	Total BMD at age 6y, g/cm^2	−0.03 [−0.05–0.01]	<0.01
	Bone fractures	0.64 [0.15–2.74]	0.54
	Enamel Permanent Defect at age 6y	2.04 [0.60–6.91]	0.25
	Enamel Primary Defect at age 6y	5.42 [0.97–30.41]	0.05
foodMEblue	Total BMC at age 6y, g	5.82 [−20.11–31.75]	0.66
	Total BMD at age 6y, g/cm^2	0.01 [−0.01–0.02]	0.49
	Bone fractures	2.77 [0.65–11.79]	0.17
	Enamel Permanent Defect at age 6y	1.72 [0.51–5.83]	0.38
	Enamel Primary Defect at age 6y	0.74 [0.19–2.89]	0.67

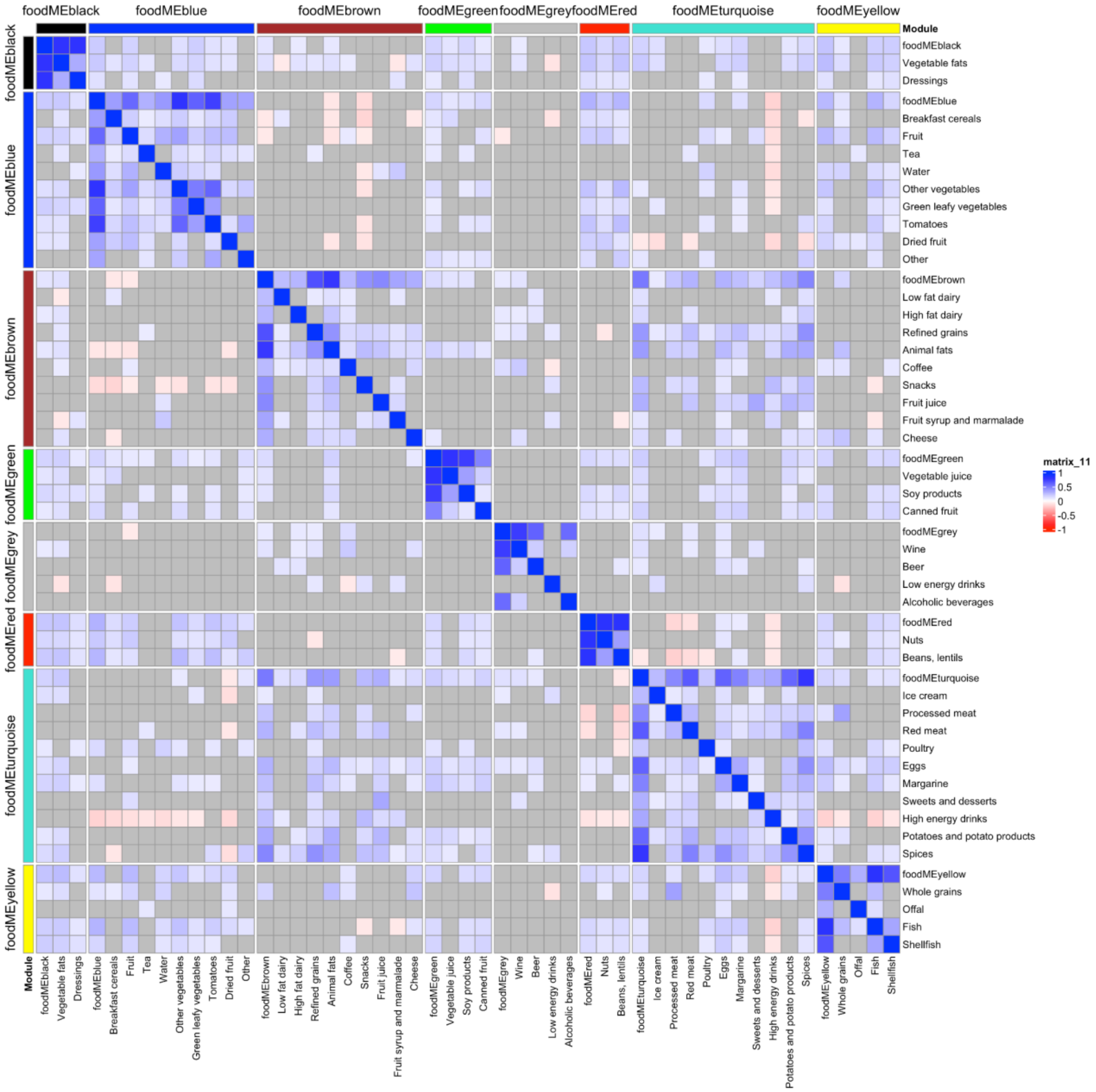

Figure S1. Heatmap of the 43 food items and 8 food modules derived from WGCNA. Correlation key: blue represents positive Pearson's correlations (p<0.05), red represents negative Pearson's correlations (p<0.05) and grey presents non-significant correlations.

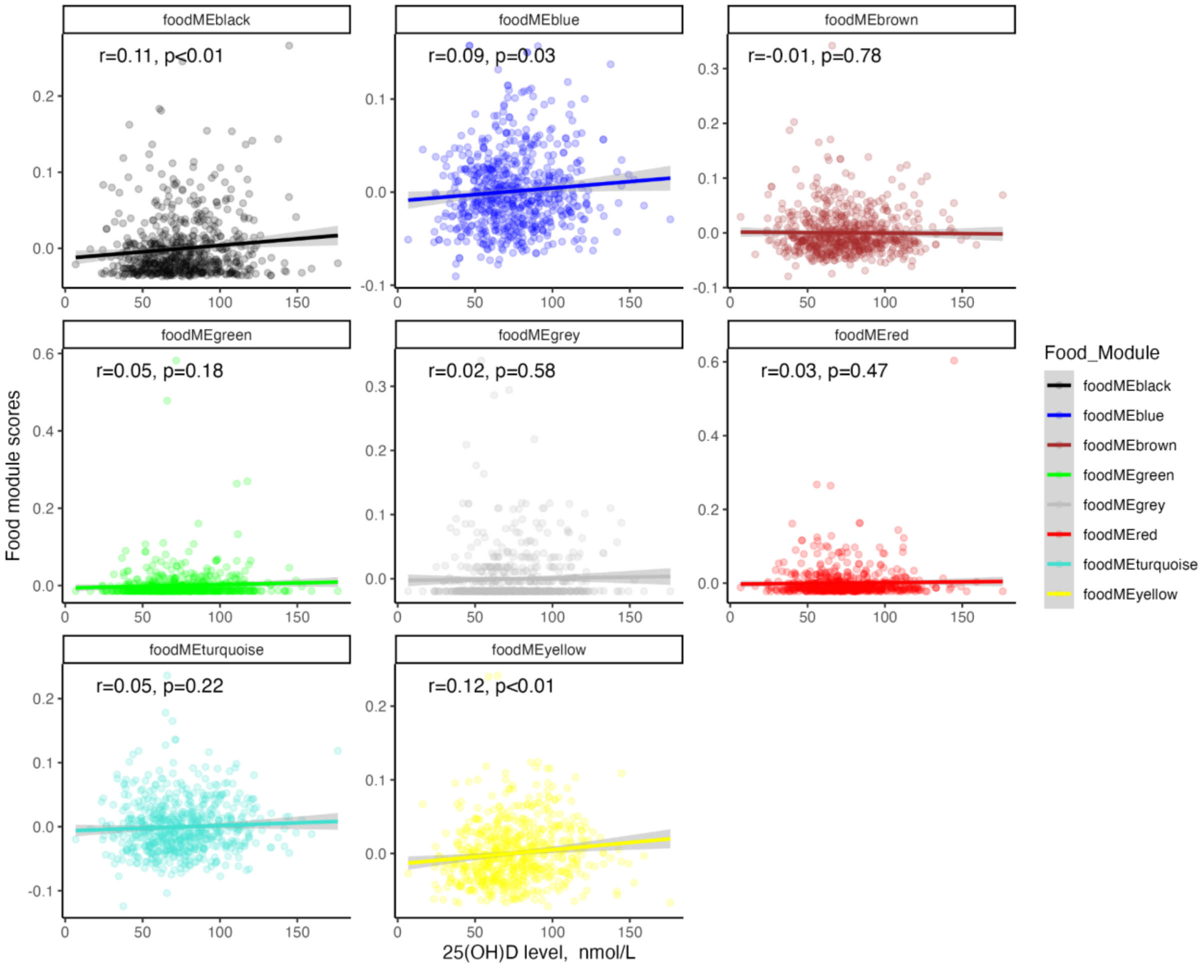

Figure S2. Figure showing correlation between 25(OH)D level at gestation week 24 and pregnancy food module scores. Pearson's partial correlation after adjusting for the season at gestation week 24 was utilized to derive correlation coefficient r and p values.
